# SARS Vaccine Protective in Mice

**DOI:** 10.3201/eid1108.041003

**Published:** 2005-08

**Authors:** Konrad Stadler, Anjeanette Roberts, Stephan Becker, Leatrice Vogel, Markus Eickmann, Larissa Kolesnikova, Hans-Dieter Klenk, Brian Murphy, Rino Rappuoli, Sergio Abrignani, Kanta Subbarao

**Affiliations:** * Chiron Vaccines, Siena, Italy;; † National Institutes of Health, Bethesda, Maryland, USA;; ‡ Institut für Virologie, Marburg, Germany

**Keywords:** coronavirus, Severe Acute Respiratory Syndrome, vaccines, inactivated, beta-Propiolactone, adjuvant, MF59

**To the Editor:** Less than a year after the identification of the severe acute respiratory syndrome coronavirus (SARS-CoV) (1), 3 independent laboratories reported protection from SARS-CoV challenge in animal models using a DNA vaccine or recombinant forms of the modified vaccinia Ankara or a parainfluenza virus, encoding the spike gene (2–4). Their protective efficacies are encouraging because they provide proof that a SARS-CoV vaccine is feasible. However, vaccines based on those technologies are not licensed for human use, and recommendation and licensing will likely take many years. We have developed an inactivated virus vaccine that induces neutralizing antibodies and protects against SARS-CoV challenge.

The vaccine was produced as described elsewhere (5). Briefly, the SARS-CoV (strain FRA, GenBank accession no. AY310120) was grown in Vero cells, inactivated with β-propiolactone (BPL), and complete inactivation was confirmed by 2 consecutive passages on Vero cells. Inactivated virus was purified by column chromatography followed by sucrose gradient centrifugation. The fraction containing virus was dialyzed against phosphate-buffered saline pH 7.2, and total protein content was determined by using the Micro BCA Protein Assay Kit (Pierce Biotechnology, Rockford, IL, USA). Immunogenicity of the vaccine was tested by immunizing BALB/c mice at 0, 2, and 4 weeks with 5 μg of inactivated virus combined with the adjuvant MF59, an oil squalene-in-water emulsion (6) approved for human use in Europe for an influenza vaccine. Ten days after the third immunization, serum samples were tested for the presence of SARS-CoV spike protein–specific antibodies by using enzyme-linked immunosorbent assay, and high titers (1–3 × 10^4^) of anti-SARS-CoV spike immunoglobulin (Ig) G antibodies were detected. IgG subclass determination indicated a predominant Th2-type immune response similar to that observed in BALB/c mice vaccinated with a UV-inactivated SARS-CoV plus alum (7).

Efficacy of the inactivated virus vaccine was also assessed in a BALB/c mouse model of SARS-CoV infection (8). Animal studies were approved by the National Institutes of Health Animal Care and Use Committee and were conducted in an animal biosafety level 3 facility. SARS-CoV replicates in the respiratory tract of BALB/c mice following intranasal infection with 10^4^ 50% tissue culture infectious doses (TCID_50_) of virus. Generally, virus titers peak within 2 days after infection and are cleared within 7 days (8). BALB/c mice were immunized at 0, 2, and 4 weeks with 5 μg of BPL-inactivated SARS virus with or without the MF59 adjuvant. Mice were also immunized with 4 different control preparations: phosphate-buffered saline, adjuvant MF59 alone, and 5 μg of BPL inactivated influenza A virus vaccine with or without MF59. Serum samples were collected 2 weeks after each dose, and assayed for their ability to neutralize SARS-CoV (8). After 2 vaccine doses, SARS-CoV neutralizing antibodies were detected only in the group of mice immunized with the BPL-inactivated SARS virus vaccine plus MF59 adjuvant (1:91). Two weeks after the third dose, the BPL-inactivated SARS virus vaccine without MF59 induced neutralizing titers of 1:64, while the adjuvanted vaccine elicited neutralizing titers >1:600 (Table).

**Table:** Immunogenicity and efficacy of β-propiolactone (BPL)–inactivated severe acute respiratory syndrome coronavirus (SARS-CoV) vaccine in mice against subsequent challenge with live SARS-CoV

Immunogen*	Neutralization titer†			Virus replication upon challenge‡			
				Lungs		Nasal turbinates	
	2 wk post 1st dose	2 wk post 2nd dose	2 wk post 3rd dose	No. infected/ no. tested	Mean (± SE) virus titer§	No. infected/ no. tested	Mean (± SE) virus titer§
PBS	<1:8	<1:8	<1:8	4/4	6.3 ± 0.3	3/4	2.8 ± 0.35
MF59	<1:8	<1:8	<1:8	4/4	6.1 ± 0.13	3/4	3.0 ± 0.58
Influenza A (5 μg)	<1:8	<1:8	<1:8	4/4	6.3 ± 0.07	3/4	2.9 ± 0.36
Influenza A (5 μg) + MF59	<1:8	<1:8	<1:8	4/4	6.0 ± 0.19	4/4	3.0 ± 0.11
BPL-SARS-CoV (5 μg)	<1:8	<1:8	1:64	1/4	2.0 ± 0.0¶#	0/4	≤1.8 ± 0**††
BPL-SARS-CoV (5 μg) + MF59	<1:8	1:91	1:645	0/4	≤1.5 ± 0¶**	0/4	≤1.8 ± 0**††

*The indicated immunogens or control preparations were administered to mice by subcutaneous injection on 3 occasions 2 weeks apart; PBS, phosphate-buffered saline. †Neutralization titers were determined as described (8). ‡Mice were challenged with 10^4^ 50% tissue culture infectious doses (TCID_50_) SARS-CoV intranasally. §Virus titers are expressed as log_10_ TCID_50_/g of tissue. ¶p<0.00001 in a 2-tailed Student *t* test, compared to titers seen in mice that were immunized with PBS. #Indicates the titer of a single animal. The remaining 3 mice had no detectable levels of virus. **Virus not detected; the lower limit of detection of infectious virus was 1.5 log_10_ TCID_50_/g in a 10% wt/vol suspension of lung homogenate and 1.8 log_10_ TCID_50_/g in a 5% wt/vol suspension of nasal turbinates. ††p = 0.025 in a 2-tailed Student *t* test, compared to titers seen in mice that were immunized with PBS.

Mice were challenged intranasally at this point with 10^4^ TCID_50_ SARS-CoV (Urbani strain, GenBank accession no. AY278741). Nasal turbinates and lung tissues were analyzed for infectious virus 2 days later (Table). SARS-CoV titers in mice from the control groups were ≈10^6^ TCID_50_ virus/g of lung tissue and ≈10^3^ TCID_50_ virus/g of nasal turbinate tissue. Complete protection from virus replication was observed in mice that received the MF59 adjuvanted SARS-CoV vaccine. Immunization with the nonadjuvanted vaccine resulted in complete protection of the upper respiratory tract and a significant reduction (30,000-fold) of viral titers in the lower respiratory tract compared to the control groups. The incomplete protection of this group was attributed to a single animal that contained detectable infectious virus in the lung.

Accelerated or enhanced virus replication or disease in immunized persons is a concern in developing any vaccine. This may be particularly true for SARS-CoV vaccines since adverse effects have been reported for one animal coronavirus vaccine, feline infectious peritonitis virus (9). Additionally, some in vitro experiments were performed with pseudotyped lentiviruses that expressed the spike glycoprotein derived from SARS-like virus isolated from civets. In these experiments, the presence of antibodies that neutralized most human isolates of SARS-CoV demonstrated enhanced entry into renal epithelial cells (10). In our studies, we did not find enhanced virus replication in the respiratory tract of vaccinated mice upon SARS-CoV challenge. However, since mice are a model of SARS-CoV infection but not disease, the issue of disease enhancement will have to be carefully evaluated if and when an appropriate animal model in which this phenomenon can be demonstrated becomes available.

In summary, an inactivated SARS-CoV vaccine, produced with a technology that has a safety record established by immunizing hundreds of millions of persons, protects mice from challenge with SARS-CoV. The vaccine adjuvanted with MF59 elicits neutralizing antibodies (titer 1:91) after only 2 doses. We conclude that the vaccine described here has desirable properties, and our data support further development and plans for clinical trials.
